# Introduction to the special research topic on the neurobiology of emotion-cognition interactions

**DOI:** 10.3389/fnhum.2014.01051

**Published:** 2015-01-30

**Authors:** Hadas Okon-Singer, Talma Hendler, Luiz Pessoa, Alexander J. Shackman

**Affiliations:** ^1^Department of Psychology, University of HaifaHaifa, Israel; ^2^Functional Brain Center, Faculty of Medicine and Sagol School of Neuroscience, School of Psychological Sciences, Wohl Institute of Advanced Imaging, Tel Aviv UniversityTel Aviv, Israel; ^3^Department of Psychology, Neuroscience and Cognitive Science Program, Maryland Neuroimaging Center, University of MarylandCollege Park, MD, USA

**Keywords:** ACC, amygdala, anxiety, attention, depression, PFC, working memory

Recent years have witnessed an explosion of interest focused on the interplay of emotion and cognition (Pessoa, 2013; Braver et al., 2014; Dolcos and Denkova, 2014). The goal of our Special Research Topic was to survey recent advances in understanding how emotional and cognitive processes interact, how they are integrated in the brain, and the implications for understanding the mind and its disorders. Investigators from across North America, Israel, and Europe contributed 19 original empirical reports as well as 15 commentaries and theoretical reviews. Their work encompasses a broad spectrum of populations and showcases a wide variety of paradigms, measures, analytic strategies, and conceptual approaches. Already (August 2014), the 34 contributions to this Special Topic have been viewed on the *Frontiers* website more than 70,000 times, shared or posted to social media networks more than 16,000 times, and cited nearly 90 times. While reading, posting, sharing, and citing are undoubtedly helpful, active debate provides a more direct means of sharpening constructs, clarifying boundary conditions, articulating unspoken assumptions, identifying soft spots in the evidentiary record, and refining models. We agree with Kenrick and Funder's suggestion that, “science best progresses through multiple and mutually critical attempts to understand the same problem. When camps with… opposing sets of biases manage to come to some level of agreement, we may be more confident of the validity of the conclusions that are agreed upon” (Kenrick and Funder, 1988, p. 32). In this regard, we were pleased to see Proudfit (Proudfit et al., 2013) and Moser (Moser et al., 2013) vigorously debate the integration of anxiety and cognitive control.

The research embodied in this Special Research Topic underscores the tremendous progress made in our understanding of emotion-cognition interactions. In particular, this work demonstrates that emotional cues and states can profoundly influence key elements of cognition, including attention (Holtmann et al., 2013; Kessel et al., 2013; Mchugo et al., 2013; Mohanty and Sussman, 2013; Morriss et al., 2013; Peers et al., 2013; Stollstorff et al., 2013), working memory (Clarke and Johnstone, 2013; Iordan et al., 2013; Robinson et al., 2013b; Stout et al., 2013; Vytal et al., 2013), cognitive control (Kalanthroff et al., 2013; Proudfit et al., 2013; Robinson et al., 2013a), reinforcement learning (Berghorst et al., 2013), and various kinds of mood-congruent information processing (van Dessel and Vogt, 2012; Harle et al., 2013; Schick et al., 2013). Several contributors provided evidence that mood can have enduring consequences for cognition (Morriss et al., 2013; Vaisvaser et al., 2013), perhaps reflecting the comparatively slow dynamics of catecholamine and hormonal neurochemistry (Sacher et al., 2013; Shansky and Lipps, 2013). These and other molecular pathways may also help to explain the impact of emotional traits on cognition (Berggren et al., 2013; Kessel et al., 2013; Moser et al., 2013; Proudfit et al., 2013).

A number of contributors provided exciting new evidence that circuits involved in attention, executive control, and working memory play a central role in emotion and emotion regulation (Aue et al., 2013; Clarke and Johnstone, 2013; Iordan et al., 2013; Peers et al., 2013; Rolls, 2013; Sheppes and Levin, 2013; Stollstorff et al., 2013). Several contributors provided evidence that putatively emotional and cognitive regions can influence one another via complex webs of connections in ways that jointly contribute to adaptive and maladaptive behavior (John et al., 2013; Morrison et al., 2013; Rolls, 2013). Taken together, this research suggests that emotion and cognition are deeply interwoven in the fabric of the brain (Dreisbach and Fischer, 2012; Crocker et al., 2013; Mcdermott et al., 2013; Moser et al., 2013; Proudfit et al., 2013; Warren et al., 2013).

Despite this progress, a number of important challenges remain. We address these challenges in more detail in the accompanying review (Okon-Singer et al., 2015). Future work aimed at developing a deeper understanding of the interplay of emotion and cognition is a matter of practical as well as theoretical importance. Many of the most common, costly, and challenging to treat neuropsychiatric disorders—anxiety, depression, schizophrenia, substance abuse, chronic pain, autism, and so on—involve prominent disturbances of both cognition *and* emotion (Millan, 2013), suggesting that they can be conceptualized as disorders of the emotional-cognitive brain (Shackman et al., in press). These disorders impose a larger burden on public health and the global economy than either cancer or cardiovascular disease (Collins et al., 2011; Diluca and Olesen, 2014; Whiteford, 2014), underscoring the importance of accelerating efforts to understand the neural systems underlying the interaction and the integration of emotion and cognition.

## Author contributions

All the authors contributed to co-editing the Special Research Topic. Hadas Okon-Singer and Alexander J. Shackman wrote the paper. All the authors edited and revised the paper.

### Conflict of interest statement

The authors declare that the research was conducted in the absence of any commercial or financial relationships that could be construed as a potential conflict of interest.
